# Motion perception: a review of developmental changes and the role of early visual experience

**DOI:** 10.3389/fnint.2015.00049

**Published:** 2015-09-15

**Authors:** Batsheva Hadad, Sivan Schwartz, Daphne Maurer, Terri L. Lewis

**Affiliations:** ^1^Department of Special Education, University of HaifaHaifa, Israel; ^2^Department of Special Education, Edmond J. Safra Brain Research Center, University of HaifaMount Carmel, Haifa, Israel; ^3^Department of Psychology, Neuroscience & Behaviour, McMaster UniversityHamilton, ON, Canada; ^4^Department of Ophthalmology and Vision Sciences, The Hospital for Sick ChildrenToronto, ON, Canada

**Keywords:** global motion, biological motion, form-from-motion, development, visual deprivation, visual experience, deprivation amblyopia, ASD autism spectrum disorders

## Abstract

Significant controversies have arisen over the developmental trajectory for the perception of global motion. Studies diverge on the age at which it becomes adult-like, with estimates ranging from as young as 3 years to as old as 16. In this article, we review these apparently conflicting results and suggest a potentially unifying hypothesis that may also account for the contradictory literature in neurodevelopmental disorders, such as Autism Spectrum Disorder (ASD). We also discuss the extent to which patterned visual input during this period is necessary for the later development of motion perception. We conclude by addressing recent studies directly comparing different types of motion integration, both in typical and atypical development, and suggest areas ripe for future research.

Interpretation of visual scenes often requires the processing of motion, for which integration of information occurs over both space and time. Psychophysical and physiological studies have distinguished between local motion processing—sensitivity to the direction of motion in a small region of the image, and global motion processing—sensitivity to the overall direction of motion in extended regions that often correspond to surfaces and objects (Braddick and Qian, 2001; Braddick et al., 2003). The perception of global motion is obtained by the integration of disparate local motion signals (Smith et al., 1994), so that, for example, an observer gets a sense of the global direction of an orchestra marching into a football stadium despite the wide range of motions created by the local motor actions of the individuals. This integration of local motion signals into a global pattern of motion is mediated by neural networks in extrastriate cortex, unlike the processing of local motion, which depends on neurons with smaller directional receptive fields in area V1 (Williams and Sekuler, 1984; Movshon et al., 1985; Smith et al., 1994; see Movshon, 1990, for a review). Specifically, global motion activates a network of areas in the dorsal stream involving primarily the MT/MST complex located on the temporo-parieto-occipital junction, and a number of extrastriate areas in relatively superior locations such as V3/V3A, V6, and areas in the intraparietal sulcus (Wattam-Bell et al., 2010).

Much of the evidence about the perception of global motion comes from lab studies using either plaid stimuli or global dot motion. Plaid stimuli are constructed from two superimposed gratings that drift in different directions (e.g., Adelson and Movshon, 1982). If the two component gratings are sufficiently similar in terms of their low level features (contrast, speed, etc.), the visual system generates the percept of a coherent single surface moving in a direction that can be different from either of the plaid's two component gratings (see Figure 1A). Global dot motion is often simulated using random-dot kinematograms (RDKs) and the random-Gabor kinematograms (RGKs), which require the perceptual system to integrate individual local motions into a global coherent motion (see Figures 1B,C, respectively). These stimuli are made up of two populations of moving dots (or Gabor patches): “signal” dots that move with a motion vector that is “coherent” over time, and “noise” dots that move in random directions (e.g., Newsome and Paré, 1988). The task is to identify the overall direction of the coherent motion. Sensitivity is typically assessed by measuring the ratio of the signal to noise dots required to accurately determine the overall direction of motion. This ratio is defined as the *Coherence threshold* and is taken to indicate the strength of motion integration. Other versions of the tasks often include detecting motion-defined form (Gunn et al., 2002; Parrish et al., 2005), discriminating coherent from incoherent motion (Reiss et al., 2005), and judging the direction of motion when the range of directions, rather than the presence of random noise, is varied (Banton et al., 1999). RDK stimuli have been used to study the development of global motion perception and its underlying mechanisms in human and non-human primates (Albright et al., 1984; De Bruyn and Orban, 1988; Born and Tootell, 1992; Smith et al., 1994; Wattam-Bell, 1994; Edwards and Badcock, 1995; Scase et al., 1998; Nakamura et al., 2003; Kiorpes and Movshon, 2004; MacKay et al., 2005; Kiorpes et al., 2006; Hess et al., 2007).

**Figure 1:**
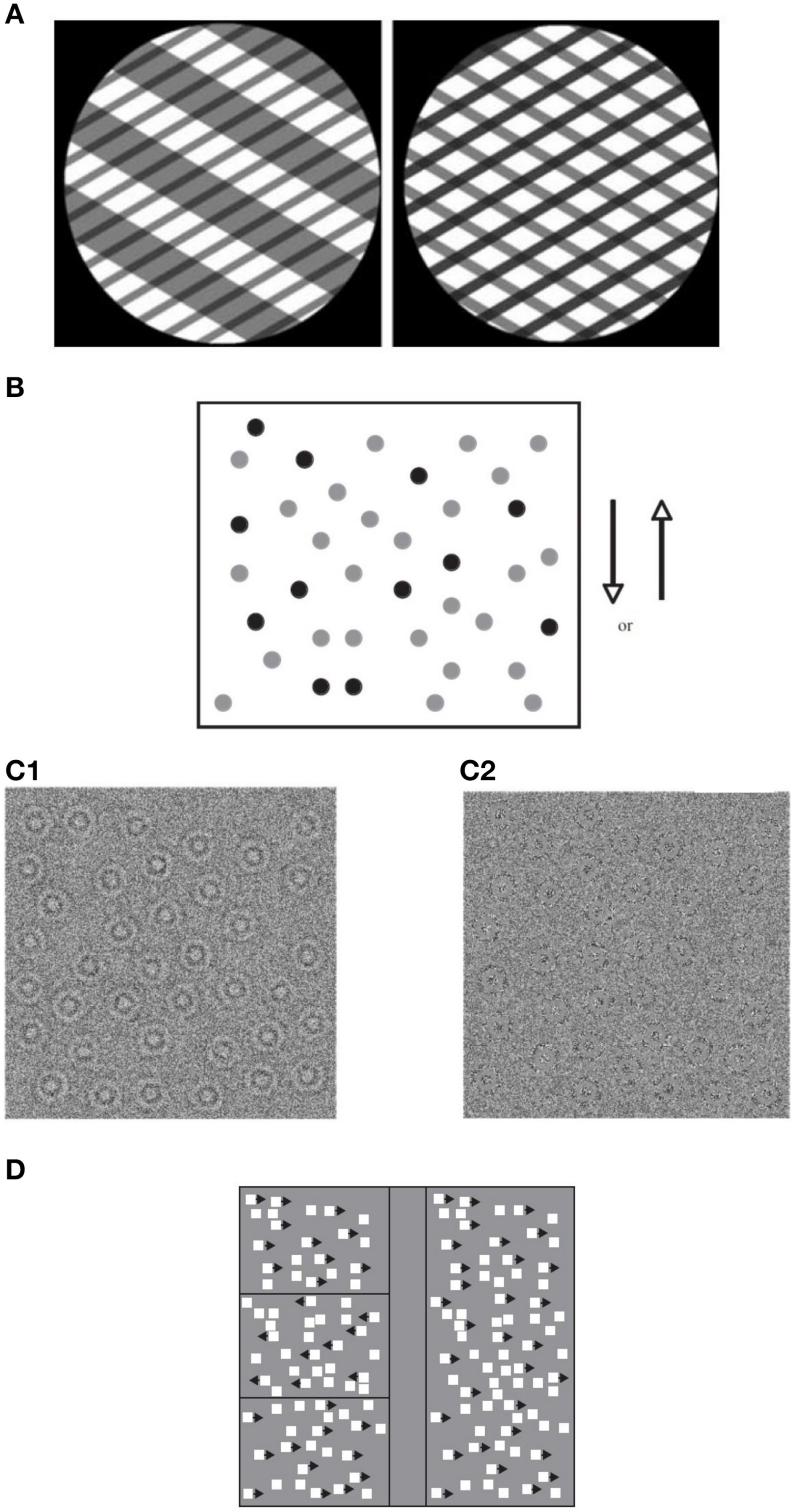
**Global motion tasks**. **(A)** Static illustration of plaid patterns composed of two superimposed square-wave grating with different orientations (shown through a circular aperture). Subject's task is to report the integrated direction of motion (adapted from Vandenbroucke et al., 2008); **(B)** Static illustration of random dot kinematogram (RDK) with 30% coherence. The illustration shows signal dots (those moving up or down) in black, and noise dots (those moving in random directions) in gray. All dots appear in black in the actual displays (adapted from Hadad et al., 2011); **(C)** Static illustration of random Gabor kinematogram (RGK) with first—**(C1)** and second—**(C2)** order motion (adapted from Ellemberg et al., 2010). **(D)** Another version of an RDK display for measuring the perception of global motion. Subject's task is to locate one of three target strips (presented on the left side of the figure) in which the signal moves in an opposite phase to those in the surrounding region (adapted from Spencer et al., 2000).

There are two special cases of motion integration that induce the perception of spatial structure: biological motion—the perception of a human figure engaged in a recognized activity (Johansson, 1973; Figure 2), and form–from-motion—the perception of the structured form defined by motion (Figure 3). In addition to global motion integration, they also depend on the spatial organization of the moving parts (e.g., Grossman and Blake, 1999). The perception of biological motion activates a network of areas in the adult extrastriate cortex involving primarily a region on the ventral bank of the occipital extent of the posterior superior-temporal sulcus (pSTS; Grossman et al., 2000), an area that receives input from both the dorsal and ventral streams (e.g., Allison et al., 2000), as well as the ventral premotor cortex (vPMC; Saygin, 2007). The perception of form–from-motion activates area KO (kinetic occipital) that is located laterally in the occipital cortex approximately 20 mm behind MT/V5 (e.g., Dupont et al., 1997).

**Figure 2:**
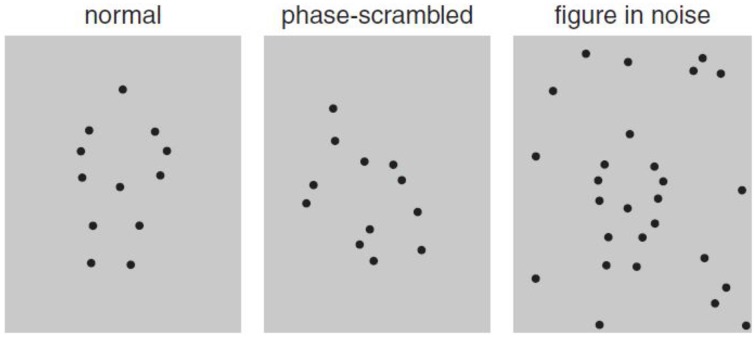
**Static illustration of biological motion displays depicting jumping (left), scrambled displays of the actor (middle), and the same biological motion embedded in noise (right; adapted from Freire et al., 2006)**.

**Figure 3:**
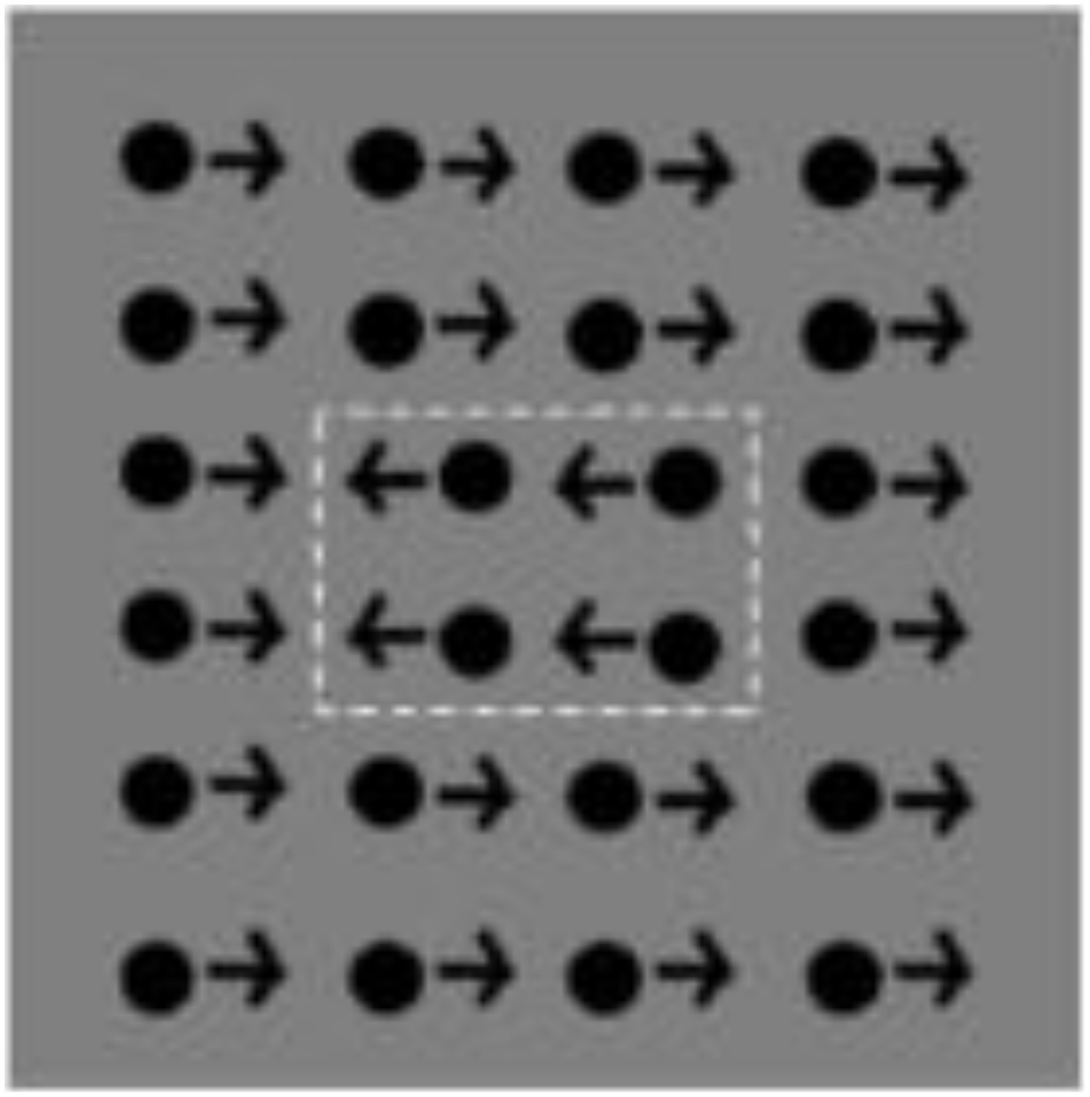
**Static illustration of form–from-motion display**. White dotted lines depict the motion-defined shape (adapted from van den Boomen et al., 2012).

The perception of motion is critical for visual development: for example, it defines the boundaries of important objects in the infants' environment, gives cues to emotional expression, and provides information about naïve physics and the location of graspable objects. Within the last two decades, numerous researchers aimed to determine the age at which these perceptual skills become adult-like. Several studies reported an early maturation for global motion while others depict a much later development, demonstrating adult-like levels only by mid-to-late childhood. The goal of this review is to examine this increasingly complex literature and to suggest ways to integrate seemingly divergent findings.

Researchers also aimed to determine the extent to which patterned visual input plays a vital role in the construction and/or preservation of the neural architecture that will later mediate motion perception. Our survey here is selective, focusing on cases of patients with abnormal early visual input caused by dense central cataracts in both eyes, and cases in which binocular input was degraded because of unilateral cataracts. Bilateral deprivation, which exemplifies the effects of visual deprivation, has often been compared to unilateral deprivation, which exemplifies the consequences not only of deprivation *per se* but also of uneven competition between the eyes. Comparing the consequences of deprivation from birth to later deprivation allows conclusions about the role of visual experience in shaping development at different ages.

We conclude by addressing recent studies directly comparing different types of motion integration that suggest avenues for a potential synthesis of this otherwise confusing literature.

## Global motion processing

### Parameters affecting global motion perception in adults

The motion signal in dot patterns is carried by spatiotemporal variations in luminance over time (i.e., “first-order” motion). The visual system is also tuned to detect motion in other stimulus characteristics such as contrast or texture (i.e., “second-order” motion; Chubb and Sperling, 1988, 1989; Cavanagh and Mather, 1989). Examples of first- and second-order motion stimuli are shown in Figure 1C. In the early stages of motion processing, first- and second-order motion appear to be analyzed by different signal processing mechanisms (Ledgeway and Smith, 1994; Nishida et al., 1997). However, it remains controversial whether, in area MT/V5, first- and second-order motion continues to be processed by different neural mechanisms (Wilson et al., 1992). The most widely accepted view is the two-stream “filter-rectify-filter” (FRF) model by Wilson that proposes that there are two motion streams. In the first, first-order motion is signaled by linear and narrowband motion energy filters. Their outputs undergo a rectifying non-linearity and are subsequently processed by a second linear filtering stage, operating at a coarse spatial scale. The intervening non-linearity has been suggested as necessary for making the second-order structure of the image accessible for further analysis carried out by the second filtering stage (Chubb and Sperling, 1988). However, the exact nature of the non-linearity is still a matter of some debate (e.g., Sperling et al., 2007).

Sensitivity to global motion in adults depends on stimulus parameters like dot density and speed. In the case of density, the reported effects are inconsistent. Some studies show that as density increases coherence thresholds decrease (Barlow and Tripathy, 1997), while others show no effect of changes in density (Eagle and Rogers, 1997; Talcott et al., 2000; Welchman and Harris, 2000; Narasimhan and Giaschi, 2012; Hutchinson et al., 2014). The reported effects of speed on global motion perception are more consistent. Higher dot speeds are often associated with greater sensitivity (e.g., Ellemberg et al., 2004; Hadad et al., 2011). There is evidence for at least two separate processing systems tuned to different ranges of speed (Anderson and Burr, 1985; Gorea et al., 1993; Hawken et al., 1994; Gegenfurtner and Hawken, 1995; Burr et al., 1998; Edwards et al., 1998; Verstraten et al., 1998; van der Smagt et al., 1999; van de Grind et al., 2001; Khuu and Badcock, 2002; Heinrich et al., 2004). The “slow” system is hypothesized to be active at speeds below 3 deg/s and the “fast” system becomes more involved as speeds increase, to an upper limit of approximately 80 deg/s (Burr et al., 1998; van de Grind et al., 2001; Khuu and Badcock, 2002).

### Developmental trajectories—from infancy to adult-like perception

As Table 1 makes clear, a growing body of data has been accumulated in recent years tracing the developmental course of motion perception. Most studies have tested first-order motion displays although the two types of motion information (first- vs. second-order) seem to differ in developmental rates^1^. Several aspects of motion processing, such as directional selectivity, seem to emerge quite early during infancy (Wattam-Bell, 1991, 1992). However, other aspects of motion processing, such as the minimum speed required to support perception of motion-defined form (Hayward et al., 2011), the maximum displacement supporting perception of movement (Parrish et al., 2005), and the discrimination of speeds (Ahmed et al., 2005; Manning et al., 2012), are not yet fully mature even at 11 years of age.

**Table 1:** **A summary of psychophysical studies on the development (from early childhood to adulthood) of global motion perception**.

**Study**	**Global motion task**	**Age groups**	**Motion direction**	**Speed (deg/s)**	**Density (dots/deg^2^)**	**Display size (in deg)**	**Dot life time**	**Trial duration**	**Luminance**	**Threshold calculation**	**Age of maturation**
Spencer et al., 2000	RDK^a^ strips	7-, 8–9-, 10–11-Year-olds (*n* = 50) and adults (*n* = 19)	Rightwards/Leftwards	5.8	4	Unspecified	Unspecified	Unspecified	Unspecified	2-Down, 1-up staircase procedure, thresholds defined as the percentage of dots moving in the same direction for 71% correct performance	10–11
Ellemberg et al., 2002	RDK (%signal)	6-Year-olds (*n* = 12) and adults (*n* = 12)	Upwards/Downwards	18	0.75	20 × 20	Limited lifetime	260	Dots—14 cd/m^2^, Background—116 cd/m^2^	2-Down, 1-up staircase procedure, thresholds defined as the percentage of Gabors moving in the same direction for 71% correct performance	6
Gunn et al., 2002	RDK^b^ (%signal) strips	4 (*n* = 37), 5 (*n* = 93), 6–7 (*n* = 60), 8–9 (*n* = 50,), 10–11-year-olds (*n* = 55) and adult (*n* = 65).	Rightwards/Leftwards	6	4	38 × 28 Monitor size	Limited life time of six frames	Up to 10,000	Unspecified	2-Down, 1-up staircase procedure, defined as the percentage of dots moving in the same direction for 71% correct performance	10–11
Atkinson et al., 2003	RDK strips	4–5.5, 5.5–6.9, 7–8, 10–11-year-olds (*n* = 140) and adults (*n* = 35)	Upwards/Downwards	5.3	4	Strip—13.4 × 6.7	limited life time of six frames	Unspecified	Unspecified	2-Down, 1-up staircase procedure, thresholds defined as the percentage of dots moving in the same direction for 71% correct performance	>5
Ellemberg et al., 2004	RGK^c^ (first and second order motion)	5-Year-olds (*n* = 24) and adults (*n* = 24).	Upwards/Downwards	1.5, 6, and 9	0.2	20 × 20	Limited lifetime	1.5 s	Mean luminance of 35 cd/m^2^	2-Down, 1-up staircase procedure, defined as the percentage of Gabors moving in the same direction for 71% correct performance	>5
Parrish et al., 2005	RDK (D_max_)	3–4, 5–6, 7–8, 9–10, 11–12-year-olds and adults. (*n* = 12)	Upward/Downward	1.2	32 Dots/deg^2^	12.8 × 9.6	Unlimited	853.6	Unspecified	Method of limits. Threshold defined as the point of maximum slope on the fitted curve, which occurs at 82%	3–4
Reiss et al., 2005	RDK (two lateral displays presented simultaneously)	4–7-Year olds (*n* = 10) and adults (*n* = 24)	Rightwards/Leftwards	2.51	Unspecified	Two displays of 13.65 × 13.65	Limited life time	Up to 6150	Dots—148.83 cd/m^2^, background—0.83 cd/m^2^	2-Down, 1-up staircase procedure, thresholds defined as the percentage of Gabors moving in the same direction for 71% correct performance	4–7
Ellemberg et al., 2010	RGK (first and second order motion, % signal	5-Year-olds (*n* = 24) and adults (*n* = 24)	Upwards/Downwards	1.5 (3 conditions): 6 arcmin/66.6 ms, 30 arcmin/333 ms, and 60 arcmin/666.6 ms	0.2	20 × 20	Limited life time	1500	Mean luminance of 35 cd/m^2^	2-Down, 1-up staircase procedure, defined as the percentage of dots moving in the same direction for 71% correct performance	>5 (For all conditions)
Hadad et al., 2011	RDK (%signal)	6–8 (*n* = 20), 9–11 (*n* = 20), 12–14 year olds (*n* = 20) and adult (*n* = 20)	Upwards/Downwards	4 and 18	0.75	17.5 × 17.5	Limited lifetime of 15 frames or 30 frames	2000	Dots—14 cd/m^2^, background—116 cd/m^2^	3-Down, 1-up adaptive staircase procedure with thresholds defined at 82% correct performance	12–14
Narasimhan and Giaschi, 2012	RDK (%signal)	5–6-Year-olds (*n* = 11) and adults (*n* = 11)	Rightwards/Leftwards	1 and 4	1, 15, and 30	7.65 × 5.57	Unspecified	400	Dots—98.5 cd/m^2^, background 1 cd/m^2^	Slope of maximum inflection on the Weibull curve fits 82% correct performance for a two-alternative forced-choice task	>6 (for all conditions)
Bogfjellmo et al., 2014	RDK (equivalent noise analysis)	6–7 (*n* = 13), 8–9 (*n* = 19), 10–11 (*n* = 20), 12–13 (*n* = 20), 14–15 (*n* = 18) and 16–17-year-olds (*n* = 13)	Clockwise/Anti clockwise	2.8 and 9.8	3.8	Diameter of 8	Limited life time of three frames	500	Mean luminance 91 cd/m^2^	2 Estimated from the EN function using QUEST	14–15 (for all conditions)
Manning et al., 2014	RDK (equivalent noise analysis)	5 (*n* = 21), 7 (*n* = 27), 9 (*n* = 25), 11-year-olds (*n* = 20) and adults (*n* = 30).	Rightwards/Leftwards	1.5	0.56	Diameter of 15	Unlimited life time	400	Dots—58.7 cd/m^2^, background—30 cd/m^2^	Single QUEST staircase tracked the minimum coherence level required for 84% correct performance.	9
Meier and Giaschi, 2014	RDK (%signal)	4–7-Year-olds (*n* = 28) and adults (*n* = 31)	Rightwards/Leftwards	30 (30 Arcmin/17 ms), 8 (23 armin/50 ms), 10 (30 armin/50 ms), 12 (38 armin/50 ms), 4 (11 arcmin/50 ms)	1.1	7.7 × 7.7	Unspecified	600	Dots—270 cd/m^2^, background—0.7 cd/m^2^	Slope of maximum inflection on the Weibull curve fits 82% correct performance for a two-alternative forced-choice task	4–7
				1 (1 arcmin/17 ms), 3 (3 armin/17 ms), 5 (5 armin/17 ms), 11 (11 armin/17 ms), 23 (23 armin/17 ms), 38 (38 armin/17 ms), 0.3 (1 armin/50 ms), 1 (3 arcmin/50 ms), 2 (5 armin/50 ms)							>7 (Group of 4–7)
Joshi and Falkenberg, 2015	RDK (%signal)	6–16-Year-olds (*n* = 119) and adults (*n* = 24)	Expansion/Contraction	1.6 and 5.5	2	Diameter of 8	Limit life time of three frames	500	Mean luminance of 50 cd/m^2^	Functional adaptive sequential testing (FAST)	>16

a *Random dot kinematogram*.

b *See Figure 1D*.

c *Random Gabor kinematogram*.

The evidence on the developmental course for sensitivity to global coherent motion is mixed. There are some early indications of this sensitivity at 11 weeks after birth (Wattam-Bell, 1994), with notable improvement in sensitivity to direction (Banton et al., 1999; Mason et al., 2003) and speed (Banton et al., 1999) after about the first 20 weeks of age. However, coherence perception does not seem to be mature even months later (Aslin and Shea, 1990; Wattam-Bell, 1990; Bertenthal and Bradbury, 1992). Studies testing this perceptual skill beyond infancy diverge on the age at which it becomes adult-like, with estimates ranging from as young as 3 years to as old as 16. Parrish et al. (2005) showed adult-like coherence thresholds in children as young as 3 years of age. Consistent with these results, Ellemberg et al. (2002) and Reiss et al. (2005) showed that thresholds exhibited by 6-year-olds children were comparable to those exhibited by adults. In contrast, Narasimhan and Giaschi (2012) and Ellemberg et al. (2004, 2010) showed that thresholds of 5- to 6-year-old children were significantly higher (poorer) than those of adults. Spencer et al. (2000) found adult-like sensitivity at the age of 10 and Gunn et al. (2002) replicated this result with an RDK paradigm and a wider age range starting at the age of 4. More recent studies demonstrated adult-like thresholds only by mid-to-late childhood. Manning et al. (2014) showed adult-like thresholds at the age of 9, Hadad et al. (2011) found that thresholds were not mature until age 13, and Bogfjellmo et al. (2014) and Joshi and Falkenberg (2015) showed maturation occurs even later (after the age of 14).

One explanation for children's immature global motion thresholds might be their relative inability to filter out motion noise (random dots) in order to decipher the motion signal (signal dots). Although this explanation might contribute to higher thresholds in children than in adults, it cannot account for the observed discrepancy across studies in the age at which global motion perception matures. There are, however, several parameters in the experimental design, stimuli, and participants characteristics that differed among the developmental studies and that are likely to account for these divergent results. Unfortunately, the relationship between each of these parameters and maturation is not obvious. In the following sections, we discuss each of these parameters in an attempt to integrate the complex body of findings.

#### Dot lifetime

The length of time that individual dots persist on the screen may well-contribute to the discrepant findings. Dot lifetimes are often limited to prevent the ability to track individual dots (e.g., Milne et al., 2002; Jackson et al., 2013). This often leads to elevated motion coherence thresholds in adults (Hiris and Blake, 1995; Festa and Welch, 1997; Braddick et al., 1998; Jackson et al., 2013). Precluding tracking strategies in this task is crucial to be sure one is measuring global motion integration and not local motion sensitivity. Discrepant results may, therefore, arise because of differences in dot lifetime across studies. Furthermore, there are other potential differences between studies limiting dot lifetime and those with dots persisting on screen for the whole trial. Short lifetimes introduce false correspondences between dots on successive frames (i.e., correspondence noise; Barlow and Tripathy, 1997)^2^, reduce the activation of motion detectors because the motion signal usually spans less than the size of a motion detector receptive field (Watamaniuk et al., 2003; Pilly and Seitz, 2009), increase the need for temporal integration (Festa and Welch, 1997), and interfere with temporal smoothness (Watamaniuk et al., 2003; Lee and Lu, 2010). Indeed, as can be seen in Table 1, most studies limiting dot lifetimes found a rather protracted developmental course for motion perception (although this critical detail is missing in some of the developmental studies). For example, Hadad et al. (2011) and Ellemberg et al. (2004, 2010), who used limited lifetime of the moving dots, found a longer developmental course than that reported by Parrish et al. (2005), who used moving dots with unlimited lifetime. Lifetime of the moving dots seems crucial also in determining deficits in motion integration in atypical development, such as in Autism Spectrum Disorder (ASD). We go back to this point later in the Developmental Disorders Section (see in Table 2).

**Table 2:** **A summary of psychophysical studies testing global motion perception in ASD using RDK**.

**Study**	**Intact/Impaired in ASD**	**Participants**	**Motion direction**	**Speed (deg/s)**	**Density (dots/deg2)**	**Dot life time**	**Trial duration (ms)**
Spencer et al., 2000	Impaired	ASD: 7–11-year-olds (*n* = 23), TD children: 7–11-year-olds (*n* = 50), TD adults (*n* = 19)	Rightwards/Leftwards	5.8	4	Limited	Unspecified
Milne et al., 2002	Impaired	ASD: 9–15-year-olds (*n* = 25), TD: 9–15-year-olds (*n* = 22)	Rightwards/Leftwards	8.8	Unspecified	Limited	1000
Pellicano et al., 2005	Impaired	ASD: 8–12-year-olds (*n* = 20), TD: 8–12-year-olds (*n* = 20)	Upwards/Downwards	Unspecified	Unspecified	Limited	600
Davis et al., 2006	Impaired only for 1000 ms	ASD: 10–18-year-olds (*n* = 9), TD: 7–15-year-olds (*n* = 9)	Rightwards/Leftwards	6.36	2.51	Unspecified	Two Conditions: 220/1000
Del Viva et al., 2006	Intact for expansion and optic flow; impaired for concentric	ASD: 6–16.6-year-olds (*n* = 13), TD: 6–19-year-olds (*n* = 31)	Rightwards–leftwards/Clockwise–anticlockwise/Circular-toward center or away from it	10	0.44	Limited	160
Milne et al., 2006	Intact	ASD: 8–13-year-olds (*n* = 23), TD: 8–12-year-olds (*n* = 23)	Rightwards/Leftwards	7	2.14	Limited	2300
Spencer and O'Brien, 2006	AS-intact; ASD-impaired	ASD: 13.5-year-olds (*n* = 15), AS^a^: 12-year-olds (*n* = 10), TD: mean age of 11.7 (*n* = 15)	Concentric	5.8	4	Limited	250
White et al., 2006	Impaired	ASD: 8–12-year-olds (*n* = 22), TD: 8-years-olds (*n* = 22)	Rightwards/Leftwards	7	2.14	Limited	2300
de Jonge et al., 2007	Intact (for all age groups)	ASD: 7–12-year-olds (*n* = 13), 13–18-year-olds (*n* = 7), adults (*n* = 9), TD: 7–12-year-olds (*n* = 11), 13–18-year-olds (*n* = 12), adults (*n* = 9)	Rightwards/Leftwards	Unspecified	Unspecified	Unspecified	Unspecified
Pellicano and Gibson, 2008	Impaired	ASD: 8–12-year-olds (*n* = 20), TD: 8–12-year-olds (*n* = 61)	Upwards/Downwards	6.33	Unspecified	Limited	600
Takarae et al., 2008	ASD with language delay-impaired; ASD without-intact	ASD with language delay: 16-year-olds (*n* = 41), ASD without language delay: 15-year-olds (*n* = 36), TD: 16.5-year-olds (*n* = 46)	Rightwards/Leftwards	3.3	2.26	Limited	300
Tsermentseli et al., 2008	ASD-impaired; AS-intact	ASD: adults (*n* = 10), AS: mean age= 23.3 (*n* = 11), TD: adults (*n* = 32)	Concentric	5.8	4	Limited	250
Atkinson, 2009	Impaired	ASD: adults (*n* = 13), TD: adults (*n* = 16)	Rightwards/Leftwards	2	6	Unspecified	200
Koldewyn et al., 2009	Impaired	ASD: 11–19-year-olds (*n* = 30), TD: 12–19-year-olds (*n* = 32)	Rightwards/Leftwards	4.5–9	Unspecified	Limited	2000
Annaz et al., 2010	Impaired	ASD: 5–12-year-olds (*n* = 23), TD: 4–12-year-olds (*n* = 34)	Rightwards/Leftwards	3.21	Unspecified	Limited	Unspecified
Jones et al., 2011	Intact	ASD: 14–16-year-olds (*n* = 89), TD: 14–16-year-olds (*n* = 52)	Rightwards/Leftwards	2.5	Varying across trails	Limited	Up to 6000
Koldewyn et al., 2011	Intact	ASD: 11–19-year-olds (*n* = 16), TD: 11–19-year-olds (*n* = 16)	Rightwards/Leftwards	4.5–9	2.2	Limited	2000
Yamasaki et al., 2011	Intact	ASD: adults (*n* = 12), TD: adults (*n* = 12)	Rightwards/Leftwards or radial outward or inward	5	0.16	Unspecified	750
Chen et al., 2012	Intact	ASD: mean age of 15.6 (*n* = 19), TD: mean age of 15.7 (*n* = 17)	Rightwards/Leftwards	5.25	5.19	Unlimited	300
Robertson et al., 2012	Impaired only in the 200 ms condition	ASD: adults (*n* = 20), TD: adults (*n* = 20)	Rightwards/Leftwards	5	1.85	limited	Three conditions: 200/400/1500
Ronconi et al., 2012	Impaired only in the central condition	ASD: 9–18-year-olds (*n* = 11), TD: 11–18-year-olds (*n* = 11)	Upward/Downward/Leftward/Rightward	12	17	Limited	300
Greimel et al., 2013	Intact	ASD: 9–16-year-olds (*n* = 17), TD: 8–15-year-olds (*n* = 17)	Rightwards/Leftwards	5	3.12	Limited	1080 ms moving randomly, 420 ms moving coherently, 1080 randomly
Koldewyn et al., 2013	Intact	ASD: 5–12-year-olds (*n* = 34), TD: 5–12-year-olds (*n* = 34)	Rightwards/Leftwards	11	0.64	Limited	100
Manning et al., 2013	Impaired only in the slow condition	ASD: 7–13-year-olds (*n* = 28), TD: 7–14-year-olds (*n* = 32)	Rightwards/Leftwards	1.5/6	0.83	Limited	1000
Manning et al., 2015	Intact	ASD: 7–13-year-olds (*n* = 31), TD: 7–13-year-olds (*n* = 31)	Rightwards/Leftwards	1.5	0.83	Limited	1000

a *Asperger syndrome*.

#### Speed

Developmental trajectories of sensitivity to motion information are affected by speed during infancy (Dannemiller and Freedland, 1989; Aslin and Shea, 1990; Wattam-Bell, 1991, 1992; Bertenthal and Bradbury, 1992; Dobkins and Teller, 1996), and later during childhood (e.g., Ellemberg et al., 2004; Ahmed et al., 2005; Narasimhan and Giaschi, 2012). It is, therefore, possible that the rather wide range of speeds used in the different studies accounts, at least in part, for the divergent findings on the age at which sensitivity to global motion reaches adult levels. However, as can be seen in in Table 1, the relation between the speed tested and developmental rates is not clear. Different studies testing overlapping speeds draw different conclusions regarding the age of maturity. Early maturation has been shown for 1.2 deg/s (Parrish et al., 2005), 2.5 deg/s (Reiss et al., 2005), and 18 deg/s (Ellemberg et al., 2002). Late maturation has been shown for 1 and 4 deg/s (Narasimhan and Giaschi, 2012), 6 deg/s (Gunn et al., 2002) and for 4 and 18 deg/s (Hadad et al., 2011).

The picture is even more complicated for the different developmental rates for slower and faster speeds within each study. Most studies report higher thresholds and larger age-related changes for slower speeds compared to faster ones (e.g., Ellemberg et al., 2004; Narasimhan and Giaschi, 2012). Narasimhan and Giaschi (2012) found higher (poorer) thresholds for the discrimination of the direction of global motion and larger age-related changes in 5-year-olds at a speed of 1 deg/s than at 4 deg/s. Similarly, Ellemberg et al. (2004) found children at this age were very immature at detecting the direction of global motion for stimuli with speeds of 1.5 deg/s, and less so at 6 and 9 deg/s. The developmental pattern is similar for thresholds to discriminate speed (Ahmed et al., 2005; Manning et al., 2012). Thresholds of 5-year-old children are immature at all speeds tested, but more so for reference speeds of 1.5 deg/s than for 6 deg/s (Ahmed et al., 2005). Similarly, children show adult-like thresholds in speed discrimination tasks at age 11 for reference speeds of 6 deg/s, but thresholds for reference speeds of 1.5 deg/s are still immature at this age (Manning et al., 2012). A similar pattern is also shown for form–from-motion. Children aged 4–6 exhibit adult-like coherence thresholds for identifying form–from motion when the elements are moving at 5 deg/s, but are immature at 0.9 deg/s and even more so at 0.1 deg/s (Hayward et al., 2011). However, Hadad et al. (2011) did not find different rates of development for random dot stimuli moving at 4 deg/s and those moving at 18 deg/s, and Manning et al. (2014) showed similar rates of development for 1.5 and 6 deg/s (although this later study found developmental rates may vary for the different speeds in terms of internal noise and sampling). One possible conclusion reconciling this group of studies is that developmental rates are similar for motion processing at intermediate and fast speeds but that processing of slower speeds, particularly those that do not fall within the optimal range of speeds processed by MT/V5 complex, develops more slowly (Manning et al., 2014).

However, the picture emerging from this literature also suggests that the two parameters defining speed–spatial offset of signal dots in an RDK (delta *x* – Δ*x*) and the temporal interval between sequential animation frames (delta *t* – Δ*t*), play a critical role in determining sensitivity to global motion (e.g., Kiorpes and Movshon, 2004; Ellemberg et al., 2010; Arena et al., 2012; Meier and Giaschi, 2014). Sensitivity to these factors in adults' motion perception has been demonstrated in detecting spatio-temporal correlation in moving two-dimensional noise patterns (van Doorn and Koenderink, 1982a,b), and in apparent motion in RDKs, which seems to occur only for relatively small spatial displacements and short interstimulus intervals (Braddick, 1974; Baker and Braddick, 1985). More recently, coherence thresholds which were measured in adults by holding either Δ*x*, Δ*t*, or speed constant while varying the other two parameters, show that larger values of Δ*x* and Δ*t* are associated with lower sensitivity than lower values, even when dots travel at the same speed (Arena et al., 2012). These two parameters seem to also affect performance during development. Wattam-Bell (1992) found that the effect of speed on age-related changes in sensitivity to motion direction during infancy is mainly related to the effects of spatial properties of the motion display, with faster development for integration across short spans. Similar effects have been shown in children. Ellemberg et al. (2010) measured coherence thresholds using RGKs in 5-year-olds and adults. Speed was held constant at 1.5 deg/s with Δ*x*/Δ*t*-values of 6 arcmin/66 ms, 30 arcmin/333 ms, and 60 arcmin/666 ms. Age-related changes were found for all displacements, but were the least for the smallest Δ*x*- and Δ*t*-values tested. Similarly, Meier and Giaschi (2014) used two Δ*t*-values in combination with seven Δ*x*-values, for a range of speeds (0.3–38 deg/s). For the longer Δ*t*, children performed as well as adults for larger Δx, and were immature for smaller Δx. When parameters were expressed as speed, there was a range of intermediate speeds (4–12 deg/s) for which maturity was dependent on the values of Δ*x*- and Δ*t*-tested.

Similar patterns have been found in developing macaques. Kiorpes and colleagues (Kiorpes and Movshon, 2004; Kiorpes et al., 2012) showed that coherence thresholds for a given speed were determined by the underlying values of Δ*x* and Δ*t*. In Kiorpes and Movshon (2004), for example, a 40-week old macaque showed optimal performance for Δ*x*-values of about 7–12 arcmin. Thresholds were best described as a function of Δ*x* rather than speed, with optimal Δ*x*-values decreasing from 15 to 40 arcmin around 3 weeks to 6–8 arcmin at about 3 years (which, at least for acuity, is known to be equivalent to ages from 3 months to 12 years in human development; Boothe et al., 1985).

#### Density

Dot density is another critical factor determining coherence thresholds both in adults (Barlow and Tripathy, 1997), and in children (Narasimhan and Giaschi, 2012). Narasimhan and Giaschi (2012) used three different densities of 1, 15, and 30 dots/deg^2^ in an RDK paradigm and showed that in children, thresholds decrease as density increases for speeds of both 1 and 4 deg/s. Density rates vary across studies, ranging from 0.2 dot/deg^2^ (Ellemberg et al., 2004, 2010), 0.57 dot/deg^2^ (Manning et al., 2014), 0.75 dot/deg^2^ (Hadad et al., 2011), 1.1 dots/deg^2^ (Meier and Giaschi, 2014), 2 dots/deg^2^ (Joshi and Falkenberg, 2015), 3.8 dots/deg^2^ (Bogfjellmo et al., 2014), 4 dots/deg^2^ (Spencer et al., 2000; Gunn et al., 2002; Atkinson et al., 2003), to 32 dots/deg^2^ (Parrish et al., 2005). This could account for the different developmental rates reported in the different studies.

However, it seems possible that these effects of density and Δ*x*, with the latter often varying unsystematically between studies as a function of speed, may both reflect the restricted range over which motion integration operates during development. The literature seems to suggest that when long-range motion information is required, as in the cases of low density and large Δ*x*, age-related changes are more robust. Furthermore, when extreme values of density are used, speed and Δ*x* become less critical in determining developmental rates. This speculative hypothesis arises from several developmental studies. Parrish et al. (2005), for example, used a speed of 1.2 deg/s and a high density rate of 32 dots/deg^2^ in an RDK paradigm and found that thresholds were adult-like by 3 years of age. The high density in this case might have compensated for the very slow speed. Consistent with this interpretation, when density is extremely low, late maturation is observed, regardless of speed. Hadad et al. (2011) used a density of 0.75 dots/deg^2^ and demonstrated late maturation of global motion with comparable age-related changes for the two speeds of 4 and 18 deg/s. Manning et al. (2014) also used a low density rate of 0.57 dots/deg^2^ and demonstrated adult-like performance at the age of 9 (and at the age of 11 for some of the aspects measured). Similar to Hadad et al. (2011), their data did not show any difference between dots moving at 1.5 and 6 deg/s, presumably because of floor effect caused by the extremely low density of the display. Bogfjellmo et al. (2014) demonstrated maturation at 14 years of age, using a density of 3.8 dots/deg^2^ (although this late maturation may also be attributed to the difficulty in making clockwise/counterclockwise discriminations and to the low contrast stimuli), and Joshi and Falkenberg (2015) used density of 2 dots/deg^2^ and found that sensitivity to radial optic flow is still immature at 16 years of age. Boot et al. (2012) used a density of 2.6 dots/deg^2^ and found adult-like performance relatively late, even when sensitivity was measured implicitly using ocular motor reaction time to fixation (Boot et al., 2012). In all of the above cases, long range interactions for motion integration are required because of low density and/or large Δ*x*, presumably leading to protracted age-related changes. This effect has also been shown in static displays such as in the case of integration of individual elements into a global contour or a shape (Kovács, 2000; Hadad and Kimchi, 2008; Hadad et al., 2010). Altogether, these findings suggest that during development integration may be restricted to a rather limited range of spatial distances, for both static and dynamic visual information.

### The role of early visual experience

Several studies have examined the effects of early visual experience on motion perception by studying amblyopic individuals who suffered anomalous visual input to one eye caused by strabismus or anisometropia (e.g., Simmers et al., 2003, 2006). Typically, these studies report thresholds that are ~4 times worse compared to controls (for second-order motion displays) in the amblyopic eye (Simmers et al., 2003) and even in the fellow eye (Simmers et al., 2003; Ho et al., 2005). Both RDKs and plaid stimuli have also been used to investigate motion perception in individuals who had been visually deprived during early infancy because of dense cataracts. These patients are of particular interest because they suffered complete pattern deprivation in the affected eye(s) until the cataracts were treated by surgically removing the natural lens of the eye and replaced with compensatory contact lenses. Patients treated for bilateral cataracts have been tested on these tasks to examine the effect of early visual deprivation on the development of motion perception. Performance of these patients has been often compared to that of patients treated for cataracts in one eye to examine the way uneven competition for cortical connections between a weaker deprived eye and a stronger fellow non-deprived eye alters the construction and/or preservation of the neural architecture that will later mediate motion perception. For lower-level visual functions, such as acuity and peripheral vision, the outcome in the deprived eye is worse after unilateral than after bilateral deprivation, unless it was offset by aggressive patching of the non-deprived eye after treatment (reviewed in Maurer and Lewis, 2013). This is the pattern also observed in physiological and anatomical studies of the visual cortex in animal models (Le Vay et al., 1980; Crawford et al., 1991). Patients in which the onset of cataracts was postnatal have been studied to identify the critical period for visual experience.

This line of research demonstrates impaired motion coherence thresholds in adults with deprivation amblyopia caused by congenital cataracts (Ellemberg et al., 2002; Constantinescu et al., 2005; Hadad et al., 2012). Regardless of the eye tested, coherence thresholds of adults with unilateral deprivation amblyopia are ~1.6 times poorer than normal (Ellemberg et al., 2002). These thresholds, measured in both the deprived and the fellow eye, are comparable to those reported for strabismic, anisometropic, and mixed amblyopes (Simmers et al., 2003). Importantly, the deficits seem independent of low-level deficits such as visual acuity and contrast sensitivity, implying an extrastriate locus for the deficit (Ellemberg et al., 2002; Constantinescu et al., 2005; Aaen-Stockdale et al., 2007).

Patients who had bilateral congenital cataracts exhibit more profound deficits in performing motion coherence tasks with thresholds in each eye being ~5 times poorer than controls (Ellemberg et al., 2002; Hadad et al., 2012). Such a surprising finding of a worse outcome after early bilateral than after early unilateral deprivation point to the detrimental effect of the absence of patterned and motion information to *both* eyes from birth on the normal development of sensitivity to global motion in either eye. Normal visual input to one eye from birth seems enough to allow the development of nearly normal sensitivity in both eyes. This pattern has also been found, although to a lesser degree, for the perception of global form, another aspect of vision involving mainly the extrastriate ventral stream (Lewis et al., 2002). Together, these findings suggest that competitive interactions between the deprived and the non-deprived eye evident in primary visual cortex co-occur with complementary interactions in at least some extrastriate areas. These complementary interactions allow a relative sparing of the neural basis mediating global motion perception after unilateral blockage of patterned visual input during early infancy.

It has been further suggested that the weaker effect of unilateral, than of bilateral, congenital deprivation, on the perception of global motion may be attributed to converging input from striate and extrastriate pathways onto binocular MT/V5 cells with large receptive fields (Maunsell and van Essen, 1983, 1987). During early unilateral deprivation, the initial development of MT/V5 cells may be driven by input from the non-deprived eye. After treatment, those cells may respond to either eye. Consistent with these suggestions is the findings that global motion is reduced slightly and equally for both the deprived and non-deprived eyes of patients treated for unilateral congenital cataract (Ellemberg et al., 2002). This suggestion that the previously deprived eye is able to drive binocular MT/V5 cells that were tuned to the direction of motion by input from the non-deprived eye is supported by recent findings from strabismic amblyopes: these patients show essentially no inter-ocular transfer of motion aftereffects for stimuli tapping the primary visual cortex but nearly normal inter-ocular transfer for global motion, which taps area MT/V5 (McColl and Mitchell, 1998).

Input from the previously deprived eye could reach area MT/V5 via cells in the primary visual cortex sensitive to low spatial frequencies that are spared after early monocular deprivation (Ellemberg et al., 1999, 2000). Another possible route of motion information to MT/V5 cells is from the pulvinar and/or other extrageniculate pathways bypassing the primary visual cortex (Rodman et al., 1990), which may play a more important role after early deprivation (Zablocka et al., 1976; Zabłocka et al., 1980) than they do after normal development (Azzopardi et al., 1998).

Comparing the performance of patients treated for congenital vs. developmental cataracts, the latter of which had clear vision during early infancy, reveals a very short sensitive period. Motion coherence thresholds do not appear to be elevated in patients treated for developmental cataracts even when the cataracts are bilateral and developed during infancy (Ellemberg et al., 2002). The normal patterned visual input that these patients receive before the visual deprivation in one or both eyes, even when given for as little as 4–8 months, allows normal coherence thresholds to develop later for the direction of global motion. This exceptionally short sensitive period appears to be specific to global motion, as the sensitive period during which normal development of other, more basic visual skills, can be damaged extends to at least mid-childhood (letter acuity, for example, is damaged by visual deprivation until at least 10 years of age (Maurer and Lewis, 2013), and the sensitive period for peripheral light sensitivity extends into adolescence; Bowering et al., 1993). Sensitive periods for other higher-order aspects of vision, such as global form or face perception, have not been tested and they, like global motion, might also have very short sensitive periods. Together, this suggests that the development of global motion mechanisms within the extrastriate visual cortex requires a short period of visual input after birth, and that some visual input to one eye is better than none.

#### Evidence from long term deprivation

Cases of late sight onset after extended periods of congenital deprivation, although very rare, also provide insights into visual development and sensitive periods in motion perception. One line of evidence comes from individuals who gained sight after an extended period of blindness, likely of congenital origin (Ostrovsky et al., 2009). The perception of motion directions seems intact in such cases; as well as the utilization of motion cues in parsing and segregation of objects. This study, however, did not include motion coherence tasks, but rather tested the role of motion cues in perceptual organization of visual objects. Nor did the authors have firm evidence that complete blindness had been present form birth. Other evidence comes from patients with a later onset of deprivation (Fine et al., 2003). M.M. who became blind at the age of 3 and gained sight 40 years later showed intact performance in many motion tasks despite severe deficits on many other visual tasks. He could successfully identify the direction of simple and complex plaid motion and perceived the barber pole illusion. Of particular relevance to motion integration skills, M.M. showed intact performance in segregating textured fields based on motion, distinguishing rotational glass motion patterns form random noise, and recognizing biological motions. M.M.'s pattern of results demonstrates a relatively short sensitive period that is consistent with the results obtained for the cataracts patients. Combined, the evidence from short and long range deprivation suggests that extended period of abnormal visual input does not necessarily preclude the development of motion integration, as long as a normal patterned visual input is received during early infancy, even for a very short period of time.

### Lessons from developmental disorders

The perception of motion is critical for visual development and therefore has been also widely studied in atypical development, such as fragile X (e.g., Kogan et al., 2004), preterm infants (e.g., MacKay et al., 2005; Atkinson and Braddick, 2007; Taylor et al., 2009), Williams syndrome (e.g., Atkinson et al., 1997, 2003, 2006; Atkinson and Braddick, 2005), dyslexia (e.g., Talcott et al., 2000; Hansen et al., 2001; Tsermentseli et al., 2008), hemiplegia (e.g., Gunn et al., 2002), dyspraxia (O'Brien et al., 2002), and ASD (e.g., Tsermentseli et al., 2008; for a review see Kaiser and Shiffrar, 2009; Simmons et al., 2009).

For some of these neurodevelopmental disorders, the critical spatial parameters affecting coherence motion have been compared to form integration, allowing some important conclusions about the underlying mechanism (e.g., Atkinson and Braddick, 2005; Milne et al., 2006; Tsermentseli et al., 2008). Based on these direct comparisons, some general conclusions about visual development have being formulated, such as the *dorsal stream vulnerability*, according to which the dorsal stream is more vulnerable to perturbations than the ventral one (Atkinson et al., 1997; MacKay et al., 2005; Atkinson et al., 2006; Atkinson and Braddick, 2007; for a review see Braddick et al., 2003; Braddick and Atkinson, 2011; but see Grinter et al., 2010, for a different perspective).

Similar to typical development, sensitivity to global motion in many of the developmental disorders is determined by spatial and temporal factors that are not always controlled across studies. In the case of ASD, for example, the contrasting reports of intact and impaired coherence perception (see in Table 2) may be related, at least in part, to dot lifetime. Most of the studies using limited lifetimes of the moving dots demonstrate impaired sensitivity to global motion (e.g., Spencer et al., 2000; Milne et al., 2002; Pellicano et al., 2005; Tsermentseli et al., 2008; Koldewyn et al., 2009). However, the other part of this literature, in which unlimited lifetime of the moving dots is employed, demonstrate intact performance in RDKs in ASD (e.g., Davis et al., 2006; Yamasaki et al., 2011; Chen et al., 2012; for the short display duration of 220 ms). It has been recently shown that typically developed individuals and those diagnosed with ASD are equally affected by the dot lifetime (Manning et al., 2015).

Contrasting reports of intact and impaired sensitivity to coherent motion in ASD may also be attributable to the spatial parameters reviewed above, such as speed (Δx) and density. However, the relationship between each of these parameters and motion sensitivity in ASD is not obvious, as these parameters are not always systematically controlled (see in Table 2). For example, similar to our argument about typical development, the effects of speed on motion coherence in ASD may in fact reflect the effect of dot spatial displacement. It is not surprising, then, that the larger deficits in ASD are shown for faster speeds (Manning et al., 2013), for which dot displacements are often large. Density of the dots may yield similar effects. In dyslexia, density has been shown to critically affect coherence thresholds (Talcott et al., 2000). Specifically, decreased sensitivity to coherent motion in dyslexia has been shown for low densities but not for high density of 12.2 dots/deg^2^. It is crucial then, to carefully control for these parameters in future attempts to study sensitivity to coherent motion and perceptual integration more generally. The mixed literature will greatly benefit from more systematic examinations of this fundamental mechanism in visual development.

## Specific cases of motion integration: Biological motion and form–from-motion

The perception of biological motion, often measured in the lab using point light animations, involves, in addition to motion integration, form–from-motion processes based on spatiotemporal integration of local motion components (see Figure 2). The precise mechanisms are still being investigated but evidence points to multiple sources of visual information. It has been shown, for example, that the perception of biological motion remains intact in patients with brain lesions that significantly impair global motion perception (Vaina et al., 1990; McLeod et al., 1996; Jokisch et al., 2005), suggesting that biological motion relies on input from both dorsal and ventral areas of the extrastriate visual cortex. Thus, comparing sensitivity to biological motion to that of global motion may provide important insights into the general mechanism of motion integration. Comparing the pattern of performance for global and biological motion to that for form–from-motion, which also involve the perception of figures depicted by spatiotemporal integration mediated by form and motion pathways, may reveal the role of the biological nature of motion in biological motion tasks vs. spatial-structural cues missing from global motion one.

The comparison across these three types of display may thus be informative. Studies providing a direct comparison of performance across the different tasks, however, are few. In the following paragraphs we point to some examples of such comparisons, both in typical and atypical development, that may provide some insights into the mechanism of motion integration.

### Developmental trajectories

There are hardly any studies providing direct comparisons of the developmental rates of the different types of motion integration. Studies focusing on biological motion demonstrate early emergence of this perceptual skill: even newborn babies show a preference for upright over inverted biological motion displays (e.g., Fox and McDaniel, 1982; Bertenthal et al., 1984; Simion et al., 2008), demonstrating their sensitivity to parameters that affect the perception of biological motion in adults (see Bertenthal et al., 1984, for a discussion). Developmental studies beyond infancy, however, show that while 5-year-olds (Pavlova et al., 2000; Blake et al., 2003) and even 4-year-olds (Sweeny et al., 2013; Zhao et al., 2014) are as sensitive as adults to biological motion in displays without noise dots, substantial age-related change is seen in this sensitivity throughout childhood when the display includes moving noise dots (Pavlova et al., 2000; Jordan et al., 2002; Freire et al., 2006). When directly compared to global motion in the same participants with dots moving at the same speed, these skills of motion integration, for both RDKs measuring global motion and biological motion, show similar, long developmental trajectories (Hadad et al., 2011).

The very few studies tracking the development of the ability to extract a figure in form–from-motion displays also report a rather wide age range (7–15 years) within which this perception reaches adult-like level (Giaschi and Regan, 1997; Schrauf et al., 1999; Gunn et al., 2002; Parrish et al., 2005). Some of these studies conducted a direct comparison between two of these three types of motion integration and only one study compared the three tasks (Reiss et al., 2005). To the best of our knowledge, however, none of these studies compared the three tasks while matching the spatial and temporal parameters, which, as shown in the first part of this review, may play a critical role in determining the developmental rates of these perceptual skills.

### The role of early visual experience

The literature on motion integration includes a variety of amblyopia sub-types; however, a number of trends emerge across studies. The three studies that have been conducted to date suggest that the perception of biological motion is preserved in anisometropic, strabismic, or mixed amblyopia, and that when poorer performance by amblyopic eyes on biological motion tasks is found, it can be attributed to general problems in signal/noise segregation or undersampling of the input, rather than a failure of motion integration. Both Neri et al. (2007) and Thompson et al. (2008) demonstrated normal inversion effects for point light stimuli in observers with strabismic and/or anisometropic amblyopia. The amblyopic eyes did exhibit elevated thresholds relative to fellow eyes and controls, but that was attributed to a greater sensitivity to the presence of noise dots rather than a selective impairment in biological motion processing (Thompson et al., 2008). Using a different task in which difficulty was controlled by removing dots from the point light displays, instead of adding noise, Luu and Levi (2013) recently demonstrated similar effects in observers with strabismic and anisometropic amblyopia. Observers had to decide whether two point light stimuli representing two dancers were moving in or out of synchrony with one another. Amblyopes exhibited sensitivity to synchronous display similar to that of the controls, indicating that biological motion processing was intact. However, both their amblyopic and their fellow eye required more signal dots than controls, presumably because of undersampling of the stimuli (Levi and Klein, 1986).

The perception of biological motion seems preserved also in patients deprived of patterned vision early in life by dense bilateral cataracts. Hadad et al. (2012) directly compared sensitivity to global motion and biological motion by testing sensitivity to both types of motion with equal speed and within the same group of patients and controls. Congenitally deprived patients exhibited normal sensitivity to biological motion, tolerating as much noise as their age-matched controls, despite the fact that these very same patients showed substantial deficits in the perception of global motion.

To determine whether the preserved sensitivity to biological motion can be attributed to the combined information from both dorsal and ventral processing streams, performance on form–from-motion displays depicting non-biological motion must also be considered. Surprisingly, performance on form–from-motion tasks requiring the detection of non-biological objects has been shown to be impaired in both the amblyopic and fellow eyes of observers with strabismic and anisometropic amblyopia (Wang et al., 2007; Hayward et al., 2011; Husk et al., 2012). These deficits cannot be attributed to visual acuity losses (Giaschi et al., 1992) or to impaired signal-noise segregation (Husk et al., 2012), and critically, have been demonstrated in the same groups of patients who showed intact performance in the global motion task. It has been proposed that abnormal second-order motion and form processing pathways (Hayward et al., 2011), or abnormal integration of form and motion (Husk et al., 2012) may underlie these deficits in form–from-motion tasks in amblyopia. However, although within-subjects comparisons of the three types of motion integration are still necessary, the preservation of biological motion perception seems mostly related to the biological nature of the task, which may recruit specialized and robust neural pathways (Vaina et al., 2001; Troje and Westhoff, 2006; Saygin, 2007; Hamm et al., 2014).

### Developmental disorders

For many of the developmental disorders for which motion integration has been studied extensively, comparing performance in the coherence motion tasks to the other specific cases of global integrations may reveal important characteristics of the underlying mechanisms. For example, in the significant controversies that have arisen over whether observers with ASD differ from typical observers in the general mechanism of motion integration, several studies demonstrated reduced sensitivity to coherent motion in RDKs (e.g., Spencer et al., 2000; Milne et al., 2002; Tsermentseli et al., 2008) but not to biological motion (e.g., Kaiser et al., 2008; Murphy et al., 2009; Rutherford and Troje, 2012). Other evidence suggests the reversed pattern: observers with ASD do not differ from typical observers in their visual sensitivity to motion in RDKs (e.g., Manning et al., 2015), but do differ from typical observers in their visual sensitivity to biological motion (e.g., Koldewyn et al., 2011). A recent study that directly compared sensitivity to global motion, biological motion, and form–from-motion, with stimulus parameters equated, suggests that the perception of biological motion may be specifically affected in ASD. Sensitivity to biological motion develops atypically even under conditions in which sensitivity to global motion (Koldewyn et al., 2011) or form–from motion (Annaz et al., 2010; but see Saygin et al., 2010) do not. If this pattern is confirmed while controlling for the critical parameters reviewed above, these results indicate that deficits cannot be generalized to a broad impairment in ASD in spatiotemporal integration, or in integration of form and motion information, but rather indicate a specific reduction in sensitivity to the animate nature of the motion that includes the specific case of human motion. A reversed pattern of spared sensitivity to biological but not to global motion observed in the case of deprivation amblyopia, may point to the role of social interactions and exposure to others' motions in the development of the ability to perceive biological motion. Alternatively, a rudimentary neural architecture sufficient to support perception of biological motion may be resistant to certain types of perturbation like visual deprivation. That alternative is supported by evidence for sensitivity to biological motion at birth, before visual experience. This specific pattern suggests that integration of local motions into an integrated human motion may recruit specialized neural pathways mediating this preserved skill in cases such as the amblyopic visual system.

## Summary and conclusions

We have summarized the developmental course of motion perception and the effects of altered visual input on the development of this visual function. Although different studies suggest different developmental rates, some important conclusions about the critical role of several factors in determining development are allowed. One possible reconciliation of this mixed literature is that developmental rates are similar for motion processing at intermediate and fast speeds but that processing of slower speeds, particularly those that do not fall within the optimal range of speeds processed by MT/V5 complex, develops more slowly. However, rather than speed, sensitivity to coherent motion throughout development may be best expressed as a function of the two parameters defining speed–spatial offset of signal dots in an RDK (Δ*x*) and the temporal interval between sequential animation frames (Δ*t*), as well as their interaction with density. This suggests that, as has been shown for shape integration in static displays (Kovács, 2000; Hadad et al., 2010), motion integration during development may be restricted to a rather limited range of spatial distances. Reviewing this literature also points to the necessity of limiting dot lifetime to preclude tracking strategies in global motion tasks in order to be sure one is measuring global motion integration and not local motion sensitivity. These often uncontrolled factors may also account for the inconsistent findings in neurodevelopmental disorders such as in ASD. It is thus crucial for future attempts to study the mechanism underlying both the normal and the abnormal development of motion integration to carefully consider these parameters.

In the second part of this review we addressed studies testing motion integration in patients with an abnormal visual history. These allow the definition of the sensitive period for development and offer some insights into its mechanism. Studies demonstrate a worse outcome after early bilateral than after early unilateral deprivation and thus point to the detrimental effect of the absence of patterned and motion information to *both* eyes from birth on the normal development of sensitivity to global motion in either eye. Studies comparing the consequences of deprivation from birth to those of later deprivation further demonstrate an exceptionally short sensitive period. The normal patterned visual input that developmental patients receive before the visual deprivation in one or both eyes, even when given for as little as 4–8 months, allows normal coherence thresholds to develop later for the direction of global motion.

Sensitivity to other specific cases of motion integration, such as biological motion, seems normal in the same group of patients. This finding, along with the other very few comparisons carried out for the different cases of motion integration, may reveal important characteristics of the mechanism underlying the perception of global motion and those underlying the perception of biological motion. Comparing global motion perception to other specific cases of motion integration while matching the critical spatial and temporal parameters noted above are of critical importance for better understanding of the mechanism underlying the development of motion integration and the way it is shaped by early visual input. Such comparisons may uncover the crucial factors for the normal development of motion integration, and may well-suggest ways by which specialized neural pathways are recruited to mediate preserved motion skills in abnormal cases such as in the amblyopic visual system.

### Conflict of interest statement

The authors declare that the research was conducted in the absence of any commercial or financial relationships that could be construed as a potential conflict of interest.

## Footnotes

^1^Studies comparing these two types of motions demonstrated larger age-related changes for second- than for first-order displays (Ellemberg et al., 2004, 2010).

^2^Dots lifetime systematically changes coherence levels so that if the lifetime is *n* frames, coherence rate is reduced by 100/*n*. Lifetime was the way motion coherence was often manipulated in classical studies (e.g., Newsome and Paré, 1988).
